# Orthorectification of Skin Nevi Images by Means of 3D Model of the Human Body

**DOI:** 10.3390/s21248367

**Published:** 2021-12-15

**Authors:** Piotr M. Szczypiński, Katarzyna Sprawka

**Affiliations:** Institute of Electronics, Lodz University of Technology, Al. Politechniki 10, 93-590 Łódź, Poland; katarzyna.sprawka@dokt.p.lodz.pl

**Keywords:** visual sensing, skin cancer, image reconstruction, whole-body model

## Abstract

Melanoma is the most lethal form of skin cancer, and develops from mutation of pigment-producing cells. As it becomes malignant, it usually grows in size, changes proportions, and develops an irregular border. We introduce a system for early detection of such changes, which enables whole-body screening, especially useful in patients with atypical mole syndrome. The paper proposes a procedure to build a 3D model of the patient, relate the high-resolution skin images with the model, and orthorectify these images to enable detection of size and shape changes in nevi. The novelty is in the application of image encoding indices and barycentric coordinates of the mesh triangles. The proposed procedure was validated with a set of markers of a specified geometry. The markers were attached to the body of a volunteer and analyzed by the system. The results of quantitative comparison of original and corrected images confirm that the orthorectification allows for more accurate estimation of size and proportions of skin nevi.

## 1. Introduction

The incidence of skin cancers among fair-skinned populations has increased over the past decades, becoming a growing public health problem [1,2]. According to [3], reports of malignant skin changes have increased by over 600% between the 1940s and 2010 worldwide. In 2020 alone, skin cancer affected over 1,500,000 new individuals, while more than 120,000 died from this condition globally [4]. Some of the skin cancer forms include squamous cell carcinoma, basal cell carcinoma, and melanoma [1]. Although melanomas constitute only around 2% of all diagnosed cases of skin cancers, they are the most deadly of all types [1,5,6]. Melanoma develops as a flat lesion from a mutation of the pigment-producing cells melanocytes. Initially, the skin spots are small, but they grow in size quite quickly and infiltrate deeper layers of the skin to finally spread to the lymph nodes, bones, and lungs if not cured. The 5-year survival rate of melanoma can reach 99% if it is diagnosed and treated at an early stage. This percentage drops sharply with the successive stages of the disease [6,7].

The primary screening method for melanoma is the total body skin examination. The dermatologist searches the skin of the patient for individual nevi. Then, one by one, nevi are examined with a dermatoscope [8]. The device is placed on the skin surface, on top of the nevi. It enables magnified visualization of the skin fragment, measurement of the nevi size, and to make a judgment on its benign or malignant form. The attributes of melanoma-evaluated nevi are known as ABCD, which stands for asymmetry, border irregularity, color, and diameter [9]. Moreover, the growth of the nevi in size, compared with the former examination result, is also used as an indicator of possible malignancy. This kind of examination is cumbersome, time-consuming, and prone to human error, especially for patients with atypical mole syndrome, who may have hundreds of nevi to examine. Automation of this procedure would facilitate monitoring of the suspected skin lesions and make the screening procedure more accessible for patients.

The solution would be to capture skin images covering the whole body of the patient. Each image would present a vast fragment of the skin, showing not a single one, but a group of nevi at the same time. Then, with the computer vision algorithms, the individual nevi would be identified, outlined, characterized in terms of their color and morphological attributes, and finally classified. However, as the image is acquired, distances between the camera lens and particular nevi vary, and the nevi are visible at different angles. As a result, the objects which are at the distance from the camera appear smaller, and the surfaces visible at the angle have distorted proportions. Therefore, such images alone do not carry sufficient information to measure the diameter or asymmetry or to enable size control in time.

**Figure 1:**
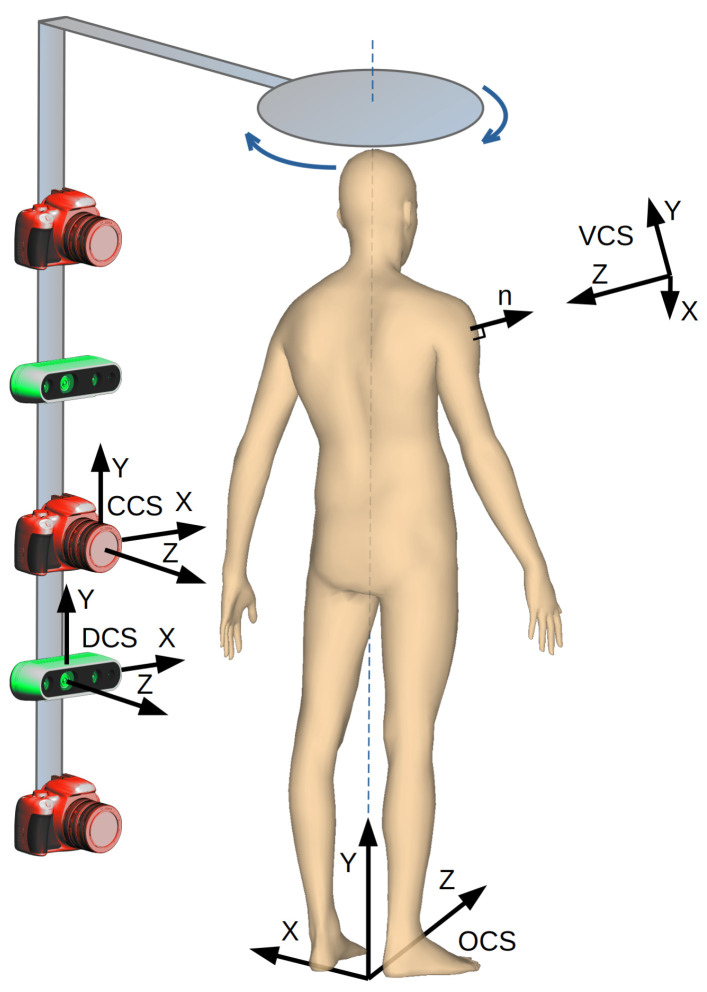
The data acquisition system with a rotating arm, cameras, and depth scanners.

Our goal is to develop a device and algorithms for fast examination of skin nevi all over the human body [10]. The analysis is to be carried out automatically. The results should be stored as a reference for the follow-up examination to be carried out after several months. The proposed solution includes a whole-human-body scanning platform (Figure 1). It consists of a rotary arm with high-resolution cameras and depth scanners mounted on it. The cameras are pointed toward the patient, and the arm rotates around the vertical axis to acquire images of, possibly, the whole body surface.

**Figure 2:**
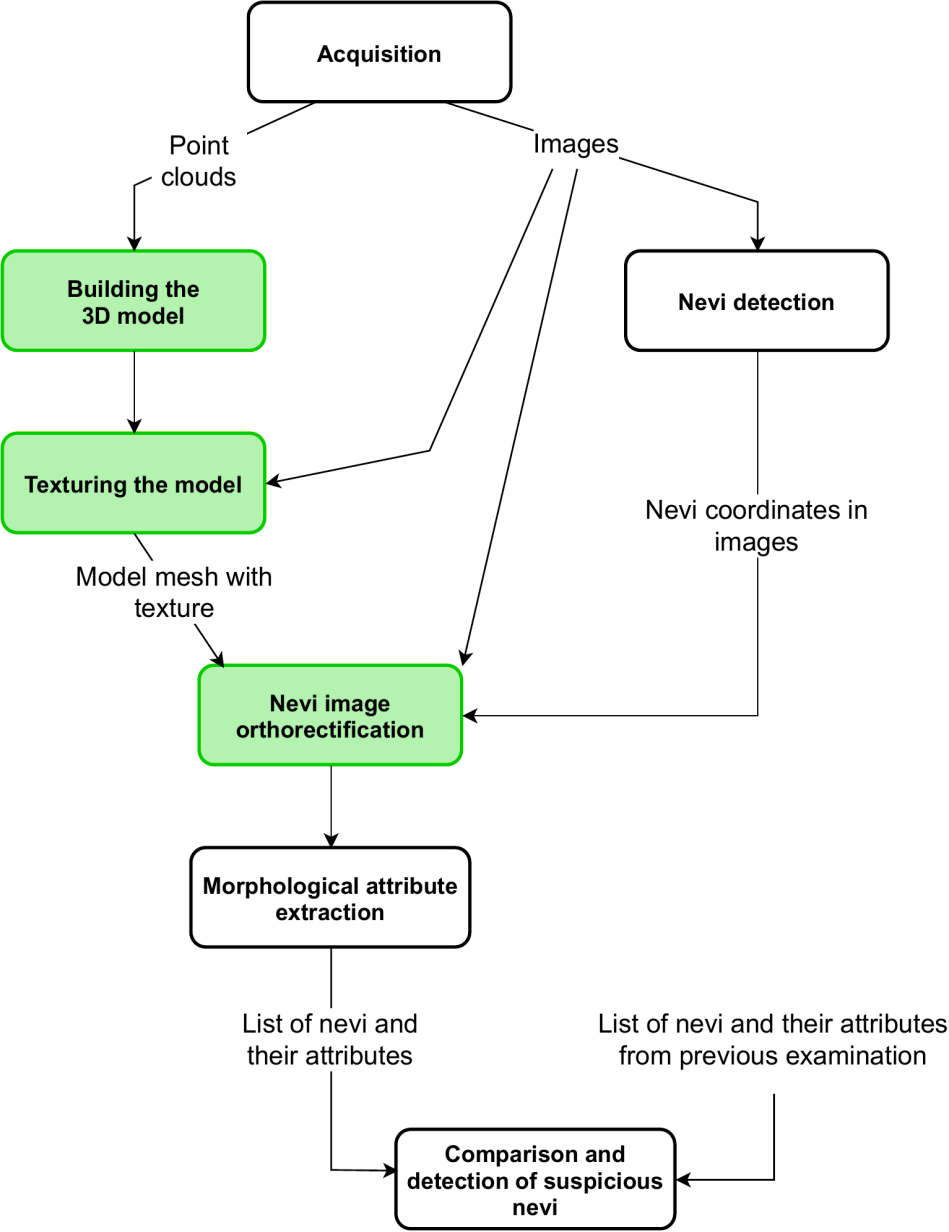
A block diagram of complete algorithm for nevi growth detection. The green background indicates algorithms discussed in this article.

The operation of the device is controlled by a computer program, which also includes the acquisition of point clouds from depth scanners and the acquisition of high-resolution images from digital cameras. Point clouds of different sides of the patient are combined to build a 3D model of the skin surface. Then, the model is covered with high-resolution images—the model is textured. During this process, the relations between the coordinates in the 3D model and the coordinates of the image fragments used for texturing are stored. The next procedure identifies the nevi in the images and generates their list with the information in which image and in what coordinates of the image the nevus was found. For each element from the list, the appropriate image fragment is geometrically corrected—orthorectified. Corrected images are used for morphological attribute extraction, such as surface area, asymmetry, or diameter of each nevus. If the result of a former examination of the patient is available, then the values of the morphological attributes of nevi are compared. If the values of the surface area or diameter increased from the previous examination, the program informs the operator about this case. This article focuses exclusively on the algorithms for building a three-dimensional model and for orthorectifying high-resolution images (Figure 2).

As already mentioned, due to the perspective effect, images of the nevi which are closer to the camera look bigger than the images of objects seen from a distance. Moreover, if a skin fragment is oriented at an angle with respect to the optical axis of the camera, the resulting image has distorted proportions. This makes it difficult to judge the actual size and shape. To reconstruct the real shape and size of the nevi, we need to know the properties of image projection onto the image sensor of the camera. In particular, we need to know the distance and the orientation angle of the skin surface in relation to the camera.

Such image reconstruction is a subject of orthophotography [11]. For decades, this technique has been applied for aerial and satellite image acquisition [12,13]. The original images are orthorectified, which means they are projected onto the topographic surface and reconstructed in the way that they were seen in frontal view with respect to this surface. The corrected image, an orthophoto, is scale uniform and can be used to measure true distances or object sizes and proportions [14,15]. This procedure is rarely applied in the correction of biomedical images. However, the need for such image correction manifests in problems of intestine unfolding [16] or in face mapping [17]. Our goal is to apply orthorectification to properly measure the sizes and characterize the shapes of skin nevi.

To retrieve the information required for the orthorectification, we use a whole-body model of the person under screening. To create the model, we use depth scanners mounted on the rotating arm similarly to the cameras. The devices have a built-in structured light source and stereo vision camera system. This arrangement enables the acquisition of depth maps, i.e., measuring the distance from the scanner to any point of the skin surface. The set of such measurements constitutes a so-called point cloud. As a result of rotation, the scanners acquire point clouds of the skin surface from various points of view. All the point clouds are, in sequence, registered and merged. The next steps involve the processing of the combined point clouds to form a surface defined by the mesh of triangles.

By having the triangle mesh model and knowing the coordinates of the camera related to the model, it is possible to establish the distances between every point of the surface and the camera. In addition, by finding the normal vector to the model’s surface and knowing the direction of the camera’s optical axis, we can find the orientation angle of the skin surface in relation to the camera. This enables the image orthorectification.

In this paper, we present methods for building a three-dimensional model of the whole human body, we describe the method of texture mapping to overlay camera images onto the surface of the triangular mesh, and we describe the algorithm for reconstruction of nevi images in their actual sizes and proportions.

## 2. Materials and Methods

### 2.1. Building a Model

The scanning procedure is as follows. The person under screening, naked or wearing underwear, stands on a platform in a designated location. The arm rotates around the patient, making a full angle (360${}^{\circ }$). During this process, the arm stops for a moment at every 45${}^{\circ }$ to acquire high-resolution images and point clouds. Therefore, the collected data present different sides of the body and cover it entirely. The angle step of 45${}^{\circ }$ was chosen to enable acquisition at eight various orientations. The orientations are dense enough to enable visualization of the whole surface of the skin, including areas under the armpits and between the legs. At the same time, the number of point clouds is small enough to build the 3D model and analyze the images within a few minutes. This way, the person under screening obtains test results in an acceptably short time.

The point clouds are sets of densely spaced three-dimensional coordinates, which are samples of the scanned surfaces. The coordinate system of each point cloud is related to the temporary location of the depth scanner whilst it was acquiring the data. The points in the cloud represent not only the surface of the human body but also surfaces of other objects within the scanner’s field of view. Therefore, the first step in point cloud processing is cropping, to keep only the points related to the human object. It is fairly simple since the space which the scanned person occupies is known and limited. The second step is to align and combine the point clouds. This requires a transformation of all the points to the common coordinate system related to the scanned object. The origin of the object coordinate system (OCS) is located at the level of the floor. The Y-axis is the rotation axis of the arm and is perpendicular to the transverse plane of the body. The Z-axis is oriented toward the front of the body and perpendicular to the coronal plane.

All the cameras and depth scanners point toward the rotation axis; each of them is mounted at the specified height hD above the floor, and their distance rD to the rotation axis is known. To transform (1) a point cloud from its own depth sensor coordinate system (DCS), the point coordinates are translated by the distance r along the Z-axis and then rotated around the Y-axis by the rotation angle of the arm at which the data were collected.

(1) $$\left[\begin{array}{c} x_{\mathrm{OCS}}\\ y_{\mathrm{OCS}}\\ z_{\mathrm{OCS}} \end{array}\right]\;=\;\left[\begin{array}{ccc} \cos(\alpha ) & 0 & -\sin(\alpha )\\ 0 & 1 & 0\\ \sin(\alpha ) & 0 & \cos(\alpha ) \end{array}\right]\left(\left[\begin{array}{c} x_{\mathrm{DCS}}\\ y_{\mathrm{DCS}}\\ z_{\mathrm{DCS}} \end{array}\right]+\left[\begin{array}{c} 0\\ h_{D}\\ -r_{D} \end{array}\right]\right)$$

After the transformation of points to OCS, their number is downsampled using a voxelized grid approach. It works by applying the 3D grid on the point cloud, bucketing the points into voxels. Next, every voxel is approximated by the spatial average of all contained points. The carefully chosen size of the voxel enables obtaining a uniform output point cloud with decimated points while preserving its fidelity. The lowered number of points results in reduced computation time and memory usage during the following processing stages. The next step is the estimation of normal vectors at every point. The applied function determines the principal axis of an arbitrary chosen number of nearest neighboring points using the covariance analysis. The resulting vector should point outside, toward the original camera location.

It must be noted that point clouds are transformed from their DCSs to OCS using a rigid transformation based on the assumption that the exact orientation of the cameras is known. This assumption is not completely true, since the devices are mounted on an arm with some inaccuracy, and the arm itself is not perfectly rigid. Moreover, the human subject moves during the acquisitions, even though we use hand supports to stabilize his or her posture. Therefore, matching the point clouds with each other and building the model is a challenging task. To solve it, we make use of state-of-the-art algorithms and their implementations in Open3D [18] and PCL [19] data processing libraries.

Since the coordinates may be inaccurate, the resulting point clouds are usually misaligned. Therefore, we apply coregistration by the iterative closest point (ICP) registration algorithm which refines the properties of the rigid transformations by minimizing the sum of point-to-point distances [20]. The ICP is a pairwise registration run in a loop for sequentially ordered pairs of point clouds and thus is prone to error. The error between the subsequent point cloud pairs propagates and may accumulate, resulting in the loop-closure problem. Therefore, an additional global optimization for loop closure was applied. Finally, the transformed point clouds are merged to form a common point cloud representing a human posture.

The next step is to remove the sparse outliers often contained in the raw data collected from depth sensors resulting from the measurement errors. They are determined by performing statistical analysis of the points with reference to their local neighborhood. A point is considered an outlier if its mean distance to its neighbors is larger than a given threshold. The threshold which was used was the standard deviation of the average distance from the neighbors. The applied value was chosen empirically and enables removing “loose points” without losing information at the edges of point clouds, which could distort the ICP procedure decreasing overlapping areas.

**Figure 3:**
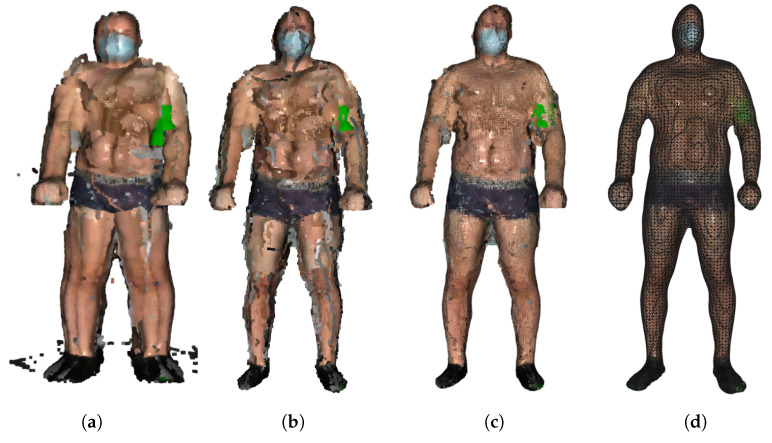
Stages of model building: (**a**) original point clouds, (**b**) registered point clouds after removal of outliers, (**c**) application of MLS algorithm, and (**d**) final model after PSR.

Still, registering may produce double wall artifacts—multiple sets of points forming close and parallel surface structures. Furthermore, the point cloud is likely to contain irregularities caused by measurement errors of the depth sensor, which appears as a wavy surface. These artifacts can be corrected and smoothed by the moving least squares (MLS) surface reconstruction algorithm, which works by locally approximating the surface with polynomials, minimizing the least squares error [21]. The smoothing effect of the MLS algorithm is presented in Figure 3.

When applying the MLS smoothing, the size of the neighborhood used for fitting has to be chosen carefully. The larger value produces a smoother surface, yet it deforms the true geometry. On the other hand, the low value can result in a weak smoothing effect. We use the neighborhood radius of 5 cm. Again, we chose this value empirically, so that the skin appears as a smooth surface, but the original geometry of the input point cloud is preserved.

**Figure 4:**
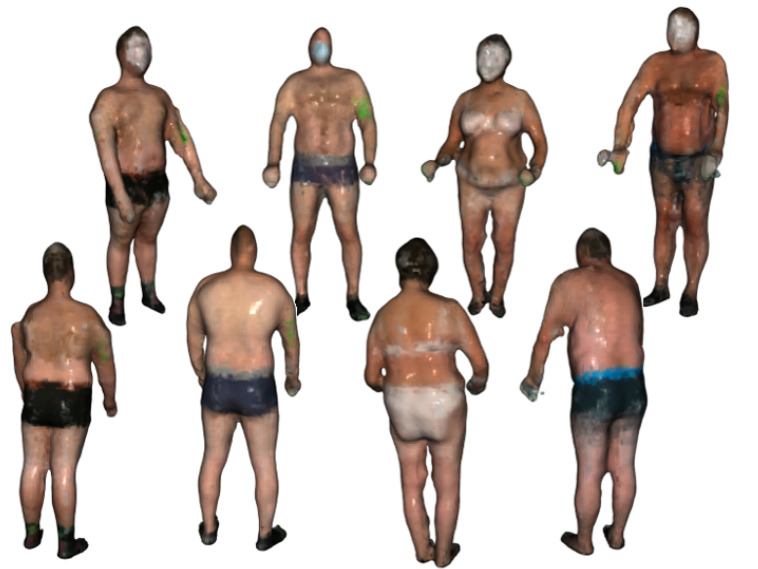
The final body models of four example volunteers.

The final step is to create a triangulated approximation (triangle mesh) of the human body surface, which is formed on the point cloud refined by the previous processing steps. We use the Poisson surface reconstruction (PSR) algorithm developed by Kazhdan et al. [22] to reconstruct the surface from so-called oriented points (points with estimated normal vectors). It produces a watertight and smooth surface. PSR applies the marching cubes algorithm for surface extraction and stores the result in an octree of a given depth. An octree of depth D produces a three-dimensional mesh of maximal resolution $2^{D}\times 2^{D}\times 2^{D}$. Thus, as the octree depth increases, mesh resolution also increases. The higher depth value results in a detailed surface; however, it also emphasizes the remaining noise and artifacts. Choosing the lower value results in greater smoothing but lower details. We chose the depth of 7 as a trade-off between smoothness and resolution. The example final models of volunteers are presented in Figure 4.

### 2.2. Texture Mapping

In principle, the models in Figure 4 define the shape of the surface. For a more complete visualization, additional information about the color is superimposed on the models. It comes from the cameras built into the depth scanner. However, this color data is of low resolution and does not allow for the identification and analysis of nevi. For identification and analysis of nevi images, high-resolution cameras are used. However, as stated before, the individual nevi visible in the images are geometrically distorted and need to be orthorectified. This process requires information from the 3D model. Therefore, correspondences between the vertices of the model mesh and the coordinates of the corresponding locations in the high-resolution images must be found. By knowing these correspondences or relations and knowing coordinates on the surface of the model, one can find the corresponding fragment of the high-resolution image. On the other hand, by selecting a point in a high-resolution image, it is possible to find a corresponding location on the surface of the model.

In computer graphics, the process of finding the relations between coordinates in raster images and coordinates of vertices in the 3D mesh is called texture mapping. The high-resolution image, the texture, is superimposed on the triangle mesh to visualize the model with appropriate detail. In the case of our approach, a known fragment of the high-resolution image, which may present an individual nevus, is matched to the coordinates on the surface of the model. Since the coordinates of the camera at the time of taking the picture are known, the distance between the camera and the location of the nevus on the model can be calculated. Moreover, the model enables determination of the normal vector to the skin surface and, thus, the orientation of this surface in relation to the optical axis of the camera. These two pieces of information are sufficient to locally orthorectify the image at the point where the nevus is visible.

In the following, we formally explain how the texture mapping is carried out, and how the relations between high-resolution images and the 3D model are used to locally orthorectify the images.

Texture mapping is a process of finding relations between the vertices of the triangle mesh ${\left[x_{\mathrm{OCS}}\;y_{\mathrm{OCS}}\;z_{\mathrm{OCS}}\right]}^{T}$ and coordinates ${\left[x_{t}y_{t}\right]}^{T}$ in the texture image. It is accomplished by transforming the coordinates of every vertex from OCS to CCS. The transformation (Equation (2)) is described by the matrix defining relative rotation of the object with respect to the arm by the angle α, and by the translation vector including camera elevation $h_{C}$ above the floor level and distance $r_{C}$ from the rotation axis. These make Equation (2) similar to Equation (1), which defines relations between DCS and OCS. However, the camera orientation may not be exactly as desired due to assembly imperfections. It can be mounted slightly askew or shifted. To account for these discrepancies, the calibration matrix $Q_{C}$ and the calibration vector $T_{C}$ are introduced into the equation. The matrix and the vector should be determined individually for each camera as a result of the calibration procedure consisting of exact alignment of the texture images with the model.

(2) $$\left[\begin{array}{c} x_{\mathrm{CCS}}\\ y_{\mathrm{CCS}}\\ z_{\mathrm{CCS}} \end{array}\right]=Q_{C}\left(\left[\begin{array}{ccc} \cos(\alpha ) & 0 & \sin(\alpha )\\ 0 & 1 & 0\\ -\sin(\alpha ) & 0 & \cos(\alpha ) \end{array}\right]\left[\begin{array}{c} x_{\mathrm{OCS}}\\ y_{\mathrm{OCS}}\\ z_{\mathrm{OCS}} \end{array}\right]-\left[\begin{array}{c} 0\\ h_{C}\\ -r_{C} \end{array}\right]\right)+T_{C}$$

After transforming the coordinates of the mesh vertices to the CCS, using the model of a pinhole camera, the vertex coordinates are projected onto the space of a two-dimensional texture image (Equation (3)). The $d_{C}$ parameter reflects the properties of the optical system of the camera and the resolution of the image sensor. As a result, we obtain coordinates in the texture image related to the vertices of the mesh.

(3) $$\left[\begin{array}{c} x_{t}\\ y_{t} \end{array}\right]\;=\;\frac{1}{d_{t}z_{\mathrm{CCS}}}\left[\begin{array}{c} x_{\mathrm{CCS}}\\ y_{\mathrm{CCS}} \end{array}\right]$$

It must be noted that there are many images of the human subject taken at various α angles and by various cameras mounted at different heights $h_{C}$ above the floor level. Therefore, the computation defined by Equations (2) and (3) has to be repeated for all available texture images individually.

There are three practical problems with texturing. The first one is the occlusion of some body fragments by the other fragments which are closer to the camera, for example, armpit areas obscured by arms. This means that the fragment of the mesh related to the occluded fragment should not be textured with the particular texture image, as the texture does not picture this fragment. Secondly, the texture should not be mapped on parts of the model that are on the opposite side—not facing the camera lens. Finally, the third difficulty is that the same regions of the skin may be visible in several images taken by different cameras or at different angles. In this case, there is a need to decide which of the images presents the fragment with the greatest accuracy and therefore which of the images is the most appropriate for texturing the related fragment of the model.

**Figure 5:**
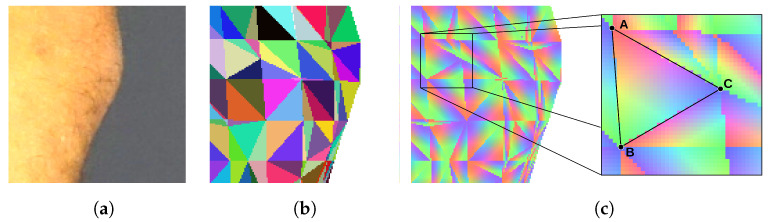
Images used to establish texture–model relations: (**a**) image of a texture, (**b**) mosaic of mesh triangles identified by unique colors, (**c**) barycentric coordinates coded by the three color components.

To determine if the particular triangle of the mesh should be textured by the particular piece of the image, we render the triangle mesh. The model is rendered as if it was visible from the point of view of the camera that has taken the texture image. Every triangle of the mesh is assigned a unique color for identification. Thus, the rendering result is an image consisting of a mosaic of colored triangles (Figure 5b). The rendered surface should exactly align with the corresponding body fragment in the texture image, and the two images should be of the same size. Therefore, the pixel of the texture image which displays a particular point of the skin can be related to the particular triangle of the mesh by checking the color of the pixel at the same location in the rendered image.

The rendered image of a triangle mosaic can be quickly produced by means of the contemporary graphics processing units (GPUs). The GPUs run the back-face culling and the occlusion culling procedures to determine which areas of the rendered surface are not visible. Therefore, the resulting image does not show triangles, which are obscured or facing away from the camera lens. As the vertices of a triangle are being projected onto the texture image, with Equations (2) and (3), we verify if the particular triangle is visible in the mosaic. If the color of the triangle in the mosaic does not match the projected one, then the particular texture is not assigned to the triangle. This way, comparison of the mosaic image with the texture image enables determination of obscured skin fragments and solves the first two difficulties.

In a multicamera system, the same triangle may be textured with information from several images obtained from various cameras or at different angles. In our approach for texturing the triangle, we select the image which presents the particular skin fragment with the highest resolution. Therefore, after projecting the triangle vertices onto the texture image, we establish the area of the projected triangle. Then, we select the image for which the area is the largest. This solves the third difficulty.

The model with superimposed images of the skin surface serves multiple purposes. It enables visualization of the human body, localization of a skin nevus on its surface, and retrieval of the information on the distance and orientation of the skin fragment. When viewing, the user can rotate or change the size of the model, and indicate a specific place on its surface, for example, a mole that he or she wants to analyze. The user should be able to immediately see the original image or orthorectified visualization of the indicated location. The other scenario is image segmentation and automatic nevi detection in texture images. After detecting the nevus in the image, it is also necessary to retrieve the information from the model needed for orthorectification.

Identification of the place indicated by the user takes place in two stages. We assume that the user observes the model at some chosen orientation and scale. This means that the textured model has been rendered in this orientation and is presented on the screen. By means of the mouse or a touch, the user indicates a particular point on the rendered image. Then, the algorithm renders the triangle mosaic image in the same scale and orientation as the model visible to the user. The algorithm checks the color of the mosaic image at the same location as indicated by the user. The color enables the identification of the corresponding triangle on the model’s mesh. This technique is known in computer graphics as mouse picking [23,24,25].

In the second scenario, the input for the procedure are coordinates of the nevus identified by image processing. Again, the mosaic of triangles is rendered from the same point of view as the texture image was taken. The color of the mosaic taken from the same coordinates enables identification of the model’s triangle.

In both scenarios, identification of the triangle is not enough. Thus, in the next step, we render an image in which each triangle is linearly shaded with RGB color components (Figure 5c). Each of the three vertices of the triangle is assigned one of the three color components. As the color over the surface of the triangle is interpolated, every point of the triangle has a unique color shade. It can be noticed that the proportions of color components at a specific location correspond with the barycentric coordinates of this location. As a result, the coordinates V of the indicated location are obtained as a sum of coordinates of the triangle vertices A, B, and C weighted by the color component proportions *r*, *g*, and *b* (Equation (4)).

(4) $$V=\frac{rA+gB+bC}{r+g+b}$$

The method explained in this section finds a link between a point in the texture image and the corresponding point on the model surface. This in turn provides data for further computation of the distance between the point and the camera and the local orientation of the surface. The method is time-efficient as it utilizes graphics processing units available on almost every computer, tablet, or smartphone, which are able to render images in a fraction of a second.

### 2.3. Image Reconstruction

Another application of the model is to obtain information about the distance and orientation of the skin surface in relation to the optical axis of the camera—the camera by which a high-resolution texture image was taken. This information is necessary to orthorectify the image from the camera. As explained in the previous section, the point indicated on the high-resolution texture image can be linked with the corresponding point and the corresponding triangle in the model’s triangle mesh.

We know the ${\left[x_{t}\;y_{t}\right]}^{T}$ coordinates of the center of a nevus. In the mosaic image, we read the color and identify the indicated triangle. From the image of the shaded triangles, we read the color components, convert them to barycentric coordinates, and calculate the coordinates of the center of the nevus in the OCS (Equation (4)). Next, we calculate the normal vector n to the surface of the triangle (Equation (5)), where A, B, and C are vectors of coordinates of the triangle vertices.

(5) $$n=\frac{\left(B-A\right)\times \left(C-A\right)}{\left|B-A\right|\;\left|C-A\right|}$$

Let us consider an imaginary orthogonal camera which serves as a means to orthorectify the image of the nevi. The virtual camera coordinate system (VCS) is created in such a way (Figure 1) that the camera is pointed at the V point. Its optical axis $Z_{\mathrm{VSC}}$ is perpendicular to the surface of the model at this point and antiparallel to the normal vector n (Equation (6)). We also assume that the $X_{\mathrm{VSC}}$ is parallel to the XZ plane of the OCS and perpendicular to the $Z_{\mathrm{VSC}}$ (Equation (7)). As the $Y_{\mathrm{VSC}}$ is perpendicular to the $X_{\mathrm{VSC}}$ and $Z_{\mathrm{VSC}}$, we calculate its axis as a vector product of the two axes (Equation (8)). Finally, we obtain Equation (9) to transform the coordinates from the OCS to the VCS.

(6) $$Z_{\mathrm{VSC}}\;=\;{\left[g_{zx}\;g_{zy}\;g_{zz}\right]}^{T}\;=\;-n$$

(7) $$X_{\mathrm{VSC}}\;=\;{\left[g_{xx}\;g_{xy}\;g_{xz}\right]}^{T}\;=\frac{{\left[g_{\mathrm{xx}}\;0\;-g_{\mathrm{zx}}\right]}^{T}}{\sqrt{g_{\mathrm{zz}}^{2}+g_{\mathrm{zx}}^{2}}}$$

(8) $$Y_{\mathrm{VSC}}\;=\;{\left[g_{yx}\;g_{yy}\;g_{yz}\right]}^{T}\;=\;X_{\mathrm{VSC}}\times Z_{\mathrm{VSC}}$$

(9) $$\left[\begin{array}{c} x_{\mathrm{VCS}}\\ y_{\mathrm{VCS}}\\ z_{\mathrm{VCS}} \end{array}\right]\;=\;G\left(\left[\begin{array}{c} x_{\mathrm{OCS}}\\ y_{\mathrm{OCS}}\\ z_{\mathrm{OCS}} \end{array}\right]-V\right)\;=\;\left[\begin{array}{ccc} g_{\mathrm{xx}} & g_{\mathrm{xy}} & g_{\mathrm{xz}}\\ g_{\mathrm{yx}} & g_{\mathrm{yy}} & g_{\mathrm{yz}}\\ g_{\mathrm{zx}} & g_{\mathrm{zy}} & g_{\mathrm{zz}} \end{array}\right]\left(\left[\begin{array}{c} x_{\mathrm{OCS}}\\ y_{\mathrm{OCS}}\\ z_{\mathrm{OCS}} \end{array}\right]-V\right)$$

By having the coordinates in the VCS, we can project them onto the surface of the sensor of the virtual camera. The equation defining orthogonal projection of the ${\left[x_{\mathrm{OCS}}\;y_{\mathrm{OCS}}\;z_{\mathrm{OCS}}\right]}^{T}$ onto the surface of the orthorectified image takes the form of Equation (10), where *s* is an arbitrary scaling factor.

(10) $$\left[\begin{array}{c} x_{g}\\ y_{g} \end{array}\right]\;=\;s\left[\begin{array}{c} x_{\mathrm{VCS}}\\ y_{\mathrm{VCS}} \end{array}\right]$$

Equations (8) and (9) enable computation of the coordinates of a point in the corrected, orthorectified image on the basis of coordinates of this point in the OCS. However, when generating the corrected image, we usually have to solve the inverse problem. While knowing the coordinates ${\left[x_{g}\;y_{g}\right]}^{T}$, we need to find coordinates on the surface of the model in the OCS and then coordinates ${\left[x_{t}\;y_{t}\right]}^{T}$ in the original texture image. To solve this problem, let us note that the matrix G in Equation (8) is orthonormal. Therefore, the inverse matrix can be simply obtained by its transposition $G^{-1}=G^{T}$. In addition, the center of the corrected image $\left({\left[x_{g}\;y_{g}\right]}^{T}={\left[0\;0\right]}^{T}\right)$ corresponds to the point V on the model’s surface. As a result of these observations, we obtain the relationship of Equation (11).

(11) $$\left[\begin{array}{c} x_{\mathrm{OCS}}\\ y_{\mathrm{OCS}}\\ z_{\mathrm{OCS}} \end{array}\right]\;=\;\frac{1}{s}\left[\begin{array}{cc} g_{\mathrm{xx}} & g_{\mathrm{yx}}\\ g_{\mathrm{xy}} & g_{\mathrm{yy}}\\ g_{\mathrm{xz}} & g_{\mathrm{yz}} \end{array}\right]\left[\begin{array}{c} x_{g}\\ y_{g} \end{array}\right]+V$$

The image reconstruction procedure is based on the fact that for each point of the reconstructed image, we find its counterpart in the texture image. For this, we use Equation (10), and then Equations (2) and (3). When coordinates are found, the color components at the point ${\left[x_{t}\;y_{t}\right]}^{T}$ are interpolated. It should be noted that changing the scaling factors enables adjustment of the resolution of the resulting image in pixels per unit length. When knowing the resolution of the corrected image, the size of the nevi in the length units can be computed.

### 2.4. Experiment

To assess the feasibility of image orthorectification with the presented method, we performed the following experiment. A volunteer with markers stuck to his skin was scanned. Every marker shows a black circle of 2 cm diameter. The volunteer was scanned using a prototype device consisting of the rotating arm with four Sony DSC RX100 cameras and three Intel RealSense D435 depth scanners. As the arm was turning, the images and point clouds were acquired at each subsequent 45 degree angle. The whole-body model was built, and the marker centers were indicated on the model. Next, the procedure for image orthorectification was applied.

**Figure 6:**
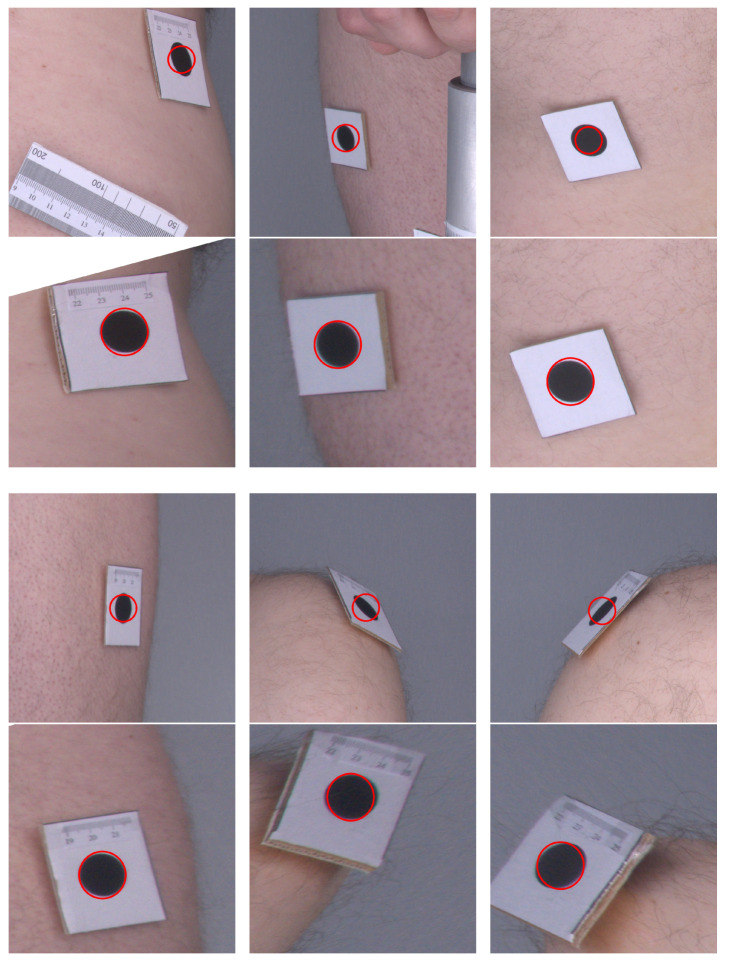
Example images before and after orthorectification. Original images are in the top rows and corrected images are in the bottom rows. Red circles indicate the expected shape of markers.

Figure 6 presents a comparison of the original images as they were acquired by the cameras and their corrected counterparts. One can see that, in the original images, the markers are elliptical in shape, and their sizes vary depending on their distance from the camera. In the corrected images, the markers are even in size and appear more similar to circles. The angles at the corners of the marker images also move closer to the right angle after applying the orthorectification procedure. Therefore, the visual inspection and comparison of the original and corrected images confirm that the orthorectification meets the expectations, and the resulting images allow for a more accurate assessment of the size and proportions.

**Figure 7:**
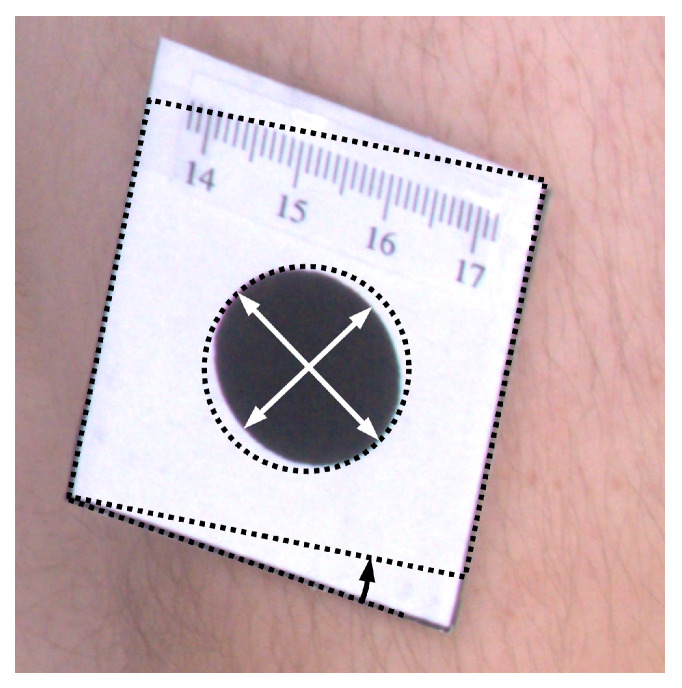
Illustration of morphological attributes measured for the assessment of the orthorectification procedure.

To quantitatively assess results of orthorectification (Figure 7), the ellipse was fitted to every marker, and proportions of semi-major to semi-minor axes were calculated. For such correctly rectified images, the proportion should be equal to one. Moreover, the area of the markers was computed, which for the circle of 2 cm diameter should be equal to 2πcm${}^{2}$. Furthermore, the shape of the tag that contains the marker is rectangular. Therefore, measuring the angle between the two edges of the tag and computing the difference between the angle and the right angle also facilitates the assessment of the rectification accuracy. The mean values of such measurements are presented in Table 1.

The resulted values of the morphological attributes are closer to the desired ones after the images were orthorectified. The shear angle was reduced more than two times. The ratio of the semi-major to semi-minor axes is closer to one, with the error of the ratio reduced 2.7 times. The standard deviations of the area computations decreased almost two times, and the estimates of the area match the expected value.

**Figure 8:**
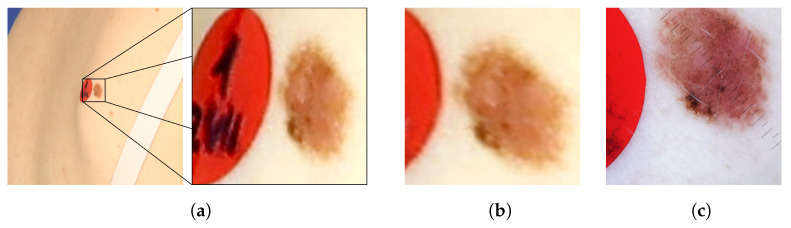
Example of a nevus: (**a**) as it appears in the original image, (**b**) the same image after orthorectification, and (**c**) the reference image acquired by means of dermatoscope.

Figure 8 presents images of an exemplary noncancerous nevus. In the original image, the nevus’s proportions are distorted. Moreover, it is impossible to judge its actual size and the proportion of length to width. In the orthorectified image, the assessment of these morphological features becomes possible. For comparison, a reference image obtained with a dermatoscope is also presented. The shape and size of the corrected and reference images are similar.

It should be noted that the results were obtained with an imperfect 3D model of the human body. The surface of the model is not as smooth as in a real human body. There are apparent errors in the orientations of the model triangle faces, which contribute to the errors in orthorectified images. Building a model that reproduces the shape of the skin more accurately would, even more, improve the outcomes of the orthorectification procedure.

## 3. Conclusions

We have presented a complete procedure for building a whole-body model to enable the correction of nevi geometries. An appropriate choice of the model reconstruction parameters enables obtaining a relatively smooth model with preserved true structure and shape of the body.

The qualitative examination shows that the models properly, though not perfectly, represent the shape of the surface of the patient’s body. The biggest problem is the patient’s movement during the scanning procedure, which results in mutually displaced surfaces that are difficult to fit. In our experiment, inaccuracies in the corrected images of markers may result from inaccuracies in the model. In addition, the source of errors is the inaccurate sticking of the markers to the skin, thus the surface of markers does not align with the surface of the skin. We are continuing work on the scanning system and model-building algorithms to minimize these errors. Moreover, as the marker centers during the experiment were indicated manually, we plan to implement image segmentation and nevi identification algorithms. This would allow for a fully automatic analysis of the entire human body surface. Following this work, we plan to verify the operation of the device and the implemented algorithms quantitatively. Taking into account the results obtained so far, we find them encouraging in confirming the feasibility to apply the proposed solution in practice.

## Figures and Tables

**Table 1:** Comparison of the resulting morphological attributes of marker images before and after orthorectification. The values in the brackets represent relative standard deviations.

	Original	Orthorectified
Angle	13.14 (0.53)	5.65 (0.21)
Ratio	1.43 (0.16)	1.16 (0.03)
Area	10.74 (0.44)	6.10 (0.23)

## Data Availability

The data presented in this study are not publicly available because optical full-body images and 3D scans of patients constitute sensitive medical information.
